# Consequences of Western and Mediterranean Diets’ Nutrients on the Microbiota–Gut–Brain Axis

**DOI:** 10.3390/nu18081258

**Published:** 2026-04-16

**Authors:** Arnaud Michel, Grégory Pourié, Tunay Kökten

**Affiliations:** Nutrition-Genetics and Exposure to Environmental Risks (NGERE), Université de Lorraine, INSERM UMRS1256, F-54000 Nancy, France; arnaud.michel5754@outlook.fr

**Keywords:** microbiota–gut–brain axis, intestinal dysbiosis, gastrointestinal diseases, neurological diseases, Western diet, Mediterranean diet

## Abstract

**Background**: The prevalence of neurodegenerative diseases like Alzheimer’s and mental disorders like depression or anxiety appears higher in patients with gastrointestinal tract diseases like inflammatory bowel disease (IBD). Conversely, depressed patients have higher rates of gastrointestinal disorders. These observations suggest bidirectional communication between the brain and the gastrointestinal tract, the so-called “gut–brain axis”. Moreover, an altered microbiota, called “dysbiosis”, has been reported in these diseases, highlighting the network between gut microbes and their host. The emergence of the microbiota as a key regulator of the gut–brain dialog has led to the establishment of the concept of the “microbiota–gut–brain axis”. **Objectives**: In this narrative review, we outline the main interaction channels between the gastrointestinal tract and the brain. Then, we summarize current knowledge of two major diets (i.e., Western and Mediterranean diets) and the principal dietary components that modulate the microbiota–gut–brain axis to discuss the mechanisms putatively involved in intestinal, psychiatric, and neurological disorders. **Conclusions**: Diet is a major factor influencing the gut microbiota, and consequently, also putatively systemic mechanisms through the microbiota–gut–brain axis. Indeed, the composition of the diet is crucial for health and disease. Despite the main role of diet, the physiological, cellular, or molecular mechanisms involved in the complex communication between the microbiome, gut, and brain are still poorly understood.

## 1. Introduction

In recent years, an increasing interest has emerged to highlight the interactions between the brain and the intestine, leading to the definition of the so-called “gut–brain axis”. Although this relationship is often presented as bidirectional communication [1], the mechanisms linking intestinal and brain functions involve the microbiota and dietary modifications, thus establishing multidirectional interactions. In recent decades, this complex but essential dialog for the health of hosts according to both physiological and pathological conditions has been studied. In a cohort of patients, Frolkis and collaborators showed that depression is associated with a significantly increased risk of developing inflammatory bowel disease (IBD) and that antidepressant treatment had an impact on the pathophysiology of IBD [2]. In addition, this study showed direct and indirect links allowing continuous multidirectional communication between the gut, the intestinal microbiota, and the brain [2]. This multiple dialog uses many physiological pathways like peripheral nerves, the endocrine system, inflammation, and various metabolites. Diet plays a major role in these multidirectional relations and can lead to systemic consequences, depending on dietary components. Indeed, the Western diet (WD)—characterized by high intakes of fat, sugar, low fiber, and processed foods—can be responsible for an alteration of the gut microbiota, which is called “dysbiosis” [3,4]. Several studies highlighted the role of dysbiosis in the development of disorders such as autism, depression, or IBD [5,6,7,8]. Inversely, the Mediterranean diet (MD)—characterized by high consumption of vegetables, fruits, nuts, legumes, and unprocessed foods—can modulate gut microbiota composition and confer benefits for host health [9,10,11,12]. Growing evidence suggests a cross-talk within the microbiota–gut–brain axis with impacts on emotions and cognitive functions through several mechanisms such as immunity, gut permeability, enteric reflex, and enteroendocrine signaling [13].

This review aims to provide a narrative analysis of the influence of diet and dietary components on the gut microbiota, intestinal and neurological health and, thus, the consequences of MD versus WD on the microbiota–gut–brain axis. Given the complexity of the field and the variability in study designs, populations, and methods, not all aspects could be covered in depth. Nevertheless, this review highlights emerging patterns, identifies key areas for future research, and provides a perspective for understanding the interactions between dietary components and the microbiota–gut–brain axis.

## 2. Methodology

The methodological approach for this narrative review involved searching PubMed, Google Scholar, and Web of Science databases using the main terms “gut–brain axis”, “Western diet”, “Mediterranean diet”, “gut-microbiota”, and “vitamins health impact”. No restrictions were placed on the publication period, as some relevant cohort studies were conducted as early as 1958. Only original studies published in English were included. Research examining this topic presents considerable heterogeneity in experimental models, methods, and outcome measures. Then, a narrative review was selected to synthesize the current evidence on the topic. The sentences were adapted to reflect a clear demonstrated causality or not in the cited studies (i.e., direct or indirect link or association), without any extrapolation.

## 3. Brain, Gut, and Intestinal Microbiota Connections

**Figure 1:**
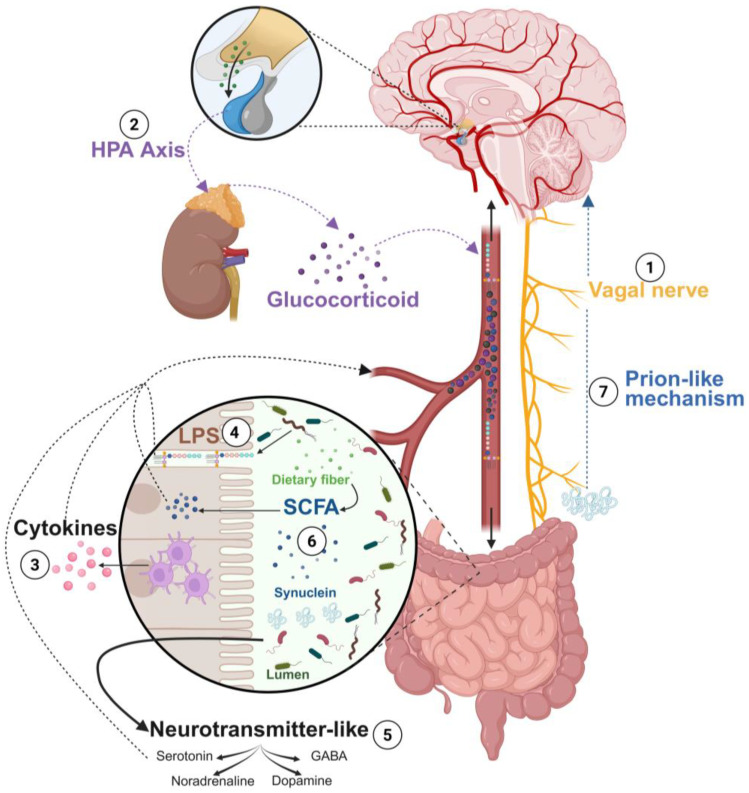
Gut–brain axis and its multidirectional communication pathways. Different types of communication pathways are involved in the gut–brain axis: the nervous system with the vagal nerve controls various intestinal functions (1); the endocrine system with the hypothalamic–pituitary–adrenal axis using glucocorticoids (2); the immune response with secretion of pro-inflammatory or anti-inflammatory cytokines by immune cells (3) in response to lipopolysaccharide (LPS) invasion, for example (4); different products from the microbiota such as neurotransmitter-like or precursors of neurotransmitters (5), local hormones, and short-chain fatty acids (SCFAs) (6); and prion-like pathways with pathogenic synuclein protein, for example (7). Created with BioRender.com (accessed on 27 March 2026).

The microbiota–gut–brain axis engages complex multidirectional communication with many pathways putatively involved, as summarized in Figure 1.

### 3.1. The Vagus Nerve Pathway

As the main component of the parasympathetic nervous system, the vagus nerve (the tenth cranial nerve) is involved in many essential functions such as digestion, mood regulation, food intake, immune response, and heart rate [14]. This nerve includes afferent and efferent branches, which play an indispensable role in the bidirectional signalization between the gut and the brain [14]. Bercik and colleagues showed that the administration of probiotics in a mouse model with chemically induced colitis is responsible for anxiety decrease, dependent on the vagus nerve, because vagotomy abolished this anxiety decrease [15]. Indeed, in an animal model of vagotomy, the major behavioral modifications initially triggered by probiotics disappeared following the surgical disruption of the vagus nerve [16]. These data highlighted the crucial role of the vagus nerve in the signal transmission between the brain and the intestine. Vagus nerve stimulation leads to acetylcholine release at the synaptic junction between nerve fiber terminals and smooth muscle by activating muscarinic receptors, ultimately responsible for muscle contraction [17]. Additionally, the vagus nerve can interact with the enteric nervous system by cholinergic activation of nicotinic receptors, responsible for bidirectional information flow [18]. Thus, as a functional approach, a few studies proposed the stimulation of the vagus nerve as a complementary treatment, not only in an experimental model of IBD [19] but also for depression in cases of human pharmacoresistance [20].

In addition to this physiological neurotransmitter crosstalk, recent studies have proposed a surprising retrograde molecular transfer from the intestine to the brain through the vagus nerve in Parkinson’s disease [21]. In this direct type of communication between the gut environment and central neurons, a pathogenic synuclein protein can be produced in the gut and progressively transferred to specific neuronal nuclei using the vagus nerve and participate in the pathogenic modification of neuronal functions [22,23]. Interestingly, such molecular transfer appears favored by bowel inflammation, leading to cell membrane destabilization [24]. Thus, this system highlights a “prion-like” mechanism and a direct route linking the brain, the gut, and the intestinal content, including the food composition and absorption, as well as intestinal microbiota [22,23].

### 3.2. The Endocrine Pathway: Hypothalamic–Pituitary–Adrenal Axis (HPA)

The hypothalamic–pituitary–adrenal (HPA) axis is a neuroendocrine system involved in many well-known physiological mechanisms, such as the cardiovascular system, immune functions, and behaviors [25,26,27]. After direct or indirect neural input by a brain region, neurons in the hypothalamic paraventricular nucleus (PVN) project to the hypothalamic median eminence, which contains hypophysiotropic neurons that produce the neurohormone corticotropin-releasing factor (CRF). Then, the CRF will bind to its receptor in the anterior part of the pituitary gland, which will lead to the release of adrenocorticotropic hormone (ACTH) into systemic circulation. Finally, after the stimulation of the melanocortin 2 receptor by ACTH in adrenal cortical producing cells, several enzymatic reactions lead to the conversion of cholesterol into corticosterone in rodents or cortisol in humans [28]. Corticosterone/cortisol is the principal endogenous glucocorticoid hormone with negative feedback in the PVN and pituitary gland [29]. Moreover, there is a crosstalk between the HPA and the gut–brain axis. Many studies in germ-free animals show the impact of intestinal microbiota on the HPA and vice versa. For example, Sudo and collaborators showed in germ-free mice a decrease in anxiety and an increase in stress response by an increase in ACTH and cortisol [30]. More recently, it has been reported in rodents that chronic stress with increased anxiety and depressive-like behavior correlates with a decrease in sucrose preference, and a modified tryptophan metabolism (i.e., the serotonin precursor) in the brain and the gut [31]. In addition, alterations in intestinal permeability and gut dysbiosis were observed, with significant modifications of microbiota as follows: an increased abundance in *Akkermansia* and *Anaerofustis* and a decrease in *Parabacteroides*, *Lachnospiraceae*, and *Ruminococcus* [31]. Finally, these authors showed a strong correlation between tryptophan metabolism, microbiota modification, and the host behaviors [31].

### 3.3. The Immune Pathway: The Role of Inflammation

The immune system and inflammation also play a major role in the gut–brain axis. External factors, such as stress or diet, can lead to gut dysbiosis and modification of the intestinal permeability, resulting in disrupted barrier integrity [32]. In these abnormal conditions, compounds produced by the intestinal microbiota, such as lipopolysaccharide (LPS), can be translocated from the gut to the systemic circulation [33]. Consequently, LPS can activate the toll-like receptor 4 (TLR4) on immune cells in the intestinal epithelium and on the microglia surface in the brain [33]. Then, the transduction of the nuclear factor-kappa B (NF-κB) pathway leads to the synthesis of pro-inflammatory cytokines, such as Interleukin-1β (IL-1β), IL-6, IL-12, and Tumor Necrosis Factor α (TNFα) [33]. These gut/brain and related systemic inflammation increase the permeability of the blood–brain barrier (BBB) and lead to neuroinflammation, with more secondary activation of the microglia and interaction between LPS and TLR4 [34]. Finally, diet-induced intestinal dysbiosis and gut inflammation can lead to neurological disease, such as psychiatric or neurodegenerative disorders, highlighting the main role of the immune pathway in the microbiota–gut–brain axis [35,36,37].

### 3.4. The Biochemical Pathway: Ammonia, Neurotransmitter-like, and Short-Chain Fatty Acid

The intestinal microbiota-produced metabolites can strongly impact physiological homeostasis. Some metabolites are rather beneficial, but others are much more deleterious. The metabolites described here are not exhaustive, but they are the most abundantly described in the literature.

Ammonia is a neurotoxic molecule produced by urease bacteria like *Helicobacter pylori* in the stomach, where it converts urea into ammonia and CO_2_ [38]. Then, in some cases, ammonia is present in too high levels in the organism, which is known as hyperammonemia [38]. This pathology leads to an alteration of the BBB integrity, an increase in intracerebral production of neurotransmitters such as dopamine and serotonin, and abnormal production of other active metabolites such as octopamine and phenylethylamine [38]. It is currently known that hyperammonemia is implicated in the onset of metabolic diseases like hepatic encephalopathy [39].

Moreover, some bacterial strains can synthesize some neurotransmitter-like molecules, such as dopamine and noradrenaline, by *Escherichia coli* [40]; serotonin by *Streptococcus thermophilus* [41]; or γ-aminobutyric acid (GABA) by *Lactobacillus reuteri* [42]. These neurotransmitters can interact with the enteric nervous system outside the control of the central nervous system (CNS) [43]. Nevertheless, it remains unclear whether neurotransmitter-like molecules can reach the CNS, especially given the difficulty of crossing the BBB without a specific transport mechanism. Microbiota can still indirectly affect the CNS by the production of a neurotransmitter precursor. Desbonnet and collaborators showed that *Bifidobacterium infantis* can lead to an increase in plasmatic tryptophan, which can impact serotonin synthesis in the brain, in a model of depression treated with probiotics [44].

Short-chain fatty acids (SCFAs), such as acetate, butyrate, and propionate, produced by anaerobic gut bacteria through saccharolytic fermentation, can interfere with the brain pathways since they can cross the BBB [45]. SCFAs play an important role in immunity homeostasis. For example, propionate and butyrate promote T regulatory cell induction, which are anti-inflammatory [46]. So, propionate and butyrate can regulate inflammation in case of excessive amounts in the intestinal mucosa [46]. Moreover, it has been shown that SCFAs protect against colitis through G protein-coupled receptor 43 (GPR43) signaling in a mouse model (i.e., this receptor is expressed on innate immune cells and modulates the resolution of inflammatory responses) [47]. Finally, SCFAs are capable of activating the sympathetic nervous system [48] and stimulating the release of serotonin by the intestinal mucosa [49]. However, a non-beneficial link between propionate and the autism-like spectrum has been described by the intraventricular administration of propionate in adult rats that showed the alteration of behaviors, cognitive deficits, and the alteration of social interactions [49]. All these results were correlated with the appearance of neuroinflammation in treated animals [49]. Another study supported this hypothesis by showing in humans an increase in fecal SCFA, like butyrate, acetate, or valeric acid, in children with autism disorder compared to the control group of children [50]. These findings highlight that the biological effects of SCFA are highly context-, tissue-, and experimental model-dependent, as they are protective within the gut and exert distinct effects in the brain.

In summary, the microbiota–gut–brain axis involves complex multidirectional communication. Many factors can modulate their interactions, whether they are intrinsic, linked to individual genetics, or extrinsic, including environmental factors such as pollution or nutrition. In Western societies, the combination of genetic predisposition to adiposity and high-caloric diets appears particularly deleterious [51].

## 4. Consequences of the Western Diet on the Microbiota–Gut–Brain Axis

**Figure 2:**
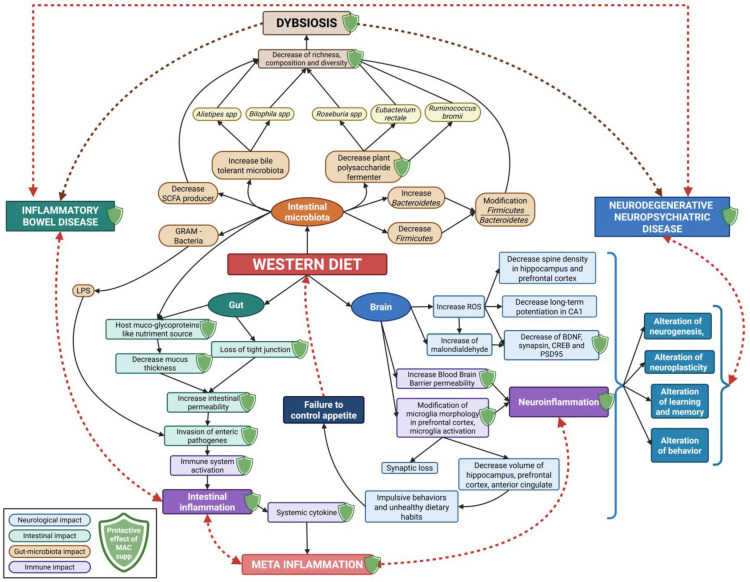
Consequences of the Western diet on the microbiota–gut–brain axis and the interest of microbiota-accessible carbohydrate (MAC) supplementation. Non-exhaustive knowledge of relations between Western diet (WD) and direct/indirect consequences on the microbiota–gut–brain axis. Consequences and interactions are numerous, since the WD promotes deleterious impacts and positive feedback loops such as amplification of the local and systemic inflammation or failure to control appetite, which contribute to the continued consumption. In parallel, the WD also promotes intestinal dysbiosis and increases intestinal permeability. Supplementation with MAC (green shield) may attenuate some of the WD effects in the gut–brain axis. The MAC’s participation is briefly presented in the conclusion. Created with BioRender.com (accessed on 27 March 2026) (ROS: reactive oxygen species, BDNF: brain-derived neurotrophic factor, CREB: cyclic AMP-responsive element-binding protein, PSD95: post-synaptic density protein 95).

The WD is a modern dietary pattern characterized by high consumption of ultra-processed, high-fat (HF), and high-sucrose food; low in fruits and vegetables; and poor in minerals and vitamins [3,4]. Consequently, people following this dietary pattern exhibit numerous deficiencies and/or physiological dysregulations [52,53,54,55,56,57]. These mechanisms are summarized in Figure 2.

### 4.1. Consequences of the Western Diet on the Gut–Microbiota

For a few decades, numerous studies in experimental models have highlighted a strong correlation between nutritional factors and modifications in the gut microbiota [58,59,60,61,62,63,64,65,66,67,68,69,70,71]. In many cases, these academic demonstrations were confirmed in human cohorts with effects on health outcomes. In experimental models, WD consumption increases the number of *Bacteroidetes* and decreases the number of *Firmicutes* [58,59]. The *Firmicutes*/*Bacteroidetes* (F/B) ratio is widely accepted to have an important influence on maintaining normal intestinal homeostasis. Increased or decreased F/B ratio is generally considered dysbiosis, which is usually observed in many diseases such as obesity, IBD, and psychiatric disorders [60]. It is currently known that *Bacteroidetes* are involved in the fermentation of non-digestible complexes, particularly those found in carbohydrate diets and host-derived glycans. Indeed, *Bacteroidetes* possess many genes implicated in carbohydrate metabolism [72]. However, more recent studies have questioned the consistency and interpretative value of the F/B ratio, as it appears highly dependent on population characteristics, methodological approaches, and disease context. Reduction in alpha-diversity, associated with compositional shifts at lower taxonomic levels, may provide more relevant information for characterizing gut dysbiosis [73,74,75]. In addition, dysbiosis was associated with an increased abundance of Gram-negative bacteria, which express LPS on their outer membrane, a molecule strongly involved in inflammation in the case of an HF diet [33,61]. Such a dietary pattern is associated with alterations in intestinal barrier integrity and may favor the translocation of LPS across the intestinal barrier, processes that are thought to promote activation of the host immune system and contribute to intestinal inflammation [33,61].

Some studies in humans have reported a correlation between a specific bacterium and intestinal inflammation. Thus, the pathobiont *Bilophila wasworthia*, a commensal Gram-negative anaerobic and bile-resistant bacterium, was first isolated from patients with appendicitis [62]. This bacterium may act synergistically with an HF diet and has been associated with intestinal barrier dysfunction and intestinal inflammation [76]. Other human studies have reported a link between modifications in the gut microbiota and metabolic pathologies; for example, a negative correlation between *Bacteroidetes* abundance and body weight [63]. Moreover, an animal-based diet leads to an increase in bile-tolerant microbiota (e.g., *Alistipes* spp., *Bilophila* spp., and *Bacteroidetes* spp.) and a decrease in plant polysaccharide fermenters like *Firmicutes* (e.g., *Roseburia* spp., *Eubacterium rectale*, and *Ruminococcus bromii*) [64]. Those shifts in intestinal microbiota composition may promote intestinal inflammation, as increases in bile-tolerant bacteria are associated with alterations in bile acid metabolism and increased production of secondary bile acids [69,70]. These secondary bile acids may directly influence the composition of the microbiota through antimicrobial activity and indirectly through interactions with membrane and nuclear receptors involved in immunity and intestinal homeostasis [69,70]. Increases in protein-degrading bacteria are associated with enhanced proteolytic fermentation in the colon, which may result in the production of metabolites such as indoles, phenols, and hydrogen sulfide (H_2_S). These metabolites have been suggested to impair intestinal epithelial barrier integrity and may contribute to immune dysregulation [71]. All these correlations suggest that diets strongly based on animal products may be associated with diseases related to intestinal inflammation [71]. However, diets rich in animal-derived products are not the only dietary patterns associated with gut microbiota dysbiosis. Carbohydrate intake has also been implicated in this phenomenon. In too high concentrations, simple sugars cannot be totally absorbed in the small intestine [65]. When consumed in excessive amounts, simple sugars can be found in the distal small intestine and colon, which leads to more substrates for bacterial growth in this region [65]. Actually, in experimental models, a high-sucrose (HS) and low-fat diet has been associated with dysbiosis, characterized by an increased abundance of *Proteobacteria* such as *Sutterella* and *Bilophila*, whose abundance has been linked to hepatic damage [66]. Similarly, combined HF and HS consumption has been reported to alter gut microbiota composition, with decreases in *Lactobacillus*, *Sphingomonas*, and *Klebsiella* and increases in *Firmicutes* such as *Faecalibacterium* and *Streptococcus* [67,68].

Finally, as a combination of HF and HS components, the WD has been widely associated with reduced diversity and richness, as well as alterations in the composition of the intestinal microbiota. These changes have been linked to potentially deleterious physiological effects, including inflammation and impaired intestinal barrier integrity [58,59,60,61,62,63,64,65,66,67,68,69,70,71]. Taken together, these observations suggest that diet-induced modifications of the gut microbiota and intestinal environment may influence the brain through molecular signaling pathways involved in microbiota–gut–brain communication. Additional evidence supporting this concept is discussed in the following paragraphs.

### 4.2. Consequences of the Western Diet on the Gut

In experimental research, quantification of specific markers is required to attest to pathogenic mechanisms. Concerning gut integrity, zonulin plays a major role in intestinal permeability, being involved in the disassembly of tight junctions. For the first time in 2000, Fasano and colleagues used zonulin as a marker of intestinal permeability [77]. Subsequently, some studies showed a strong concentration of zonulin in the serum of obese patients [78,79,80,81], suggesting altered intestinal permeability. Moreover, the level of zonulin is dependent on microbiota richness and composition and, consequently, on the type of food supply, with evidence indicating that higher intakes of fiber, n-3 polyunsaturated fatty acids, and micronutrients are associated with lower serum zonulin concentrations, suggesting improved intestinal permeability, whereas dietary patterns low in these components, such as WD, may be linked to impaired intestinal barrier function [82]. A study of 102 women showed that an HF diet increased BMI and serum zonulin, not associated with microbiota diversity but responsible for altering butyrate production, which regulates intestinal permeability [83]. Consequently, with the WD, an alteration of the intestinal barrier integrity is noted, which induces activation of the immune system by several mechanisms and subsequent inflammation.

Moreover, in an experimental model, a dietary fiber deficiency leads the intestinal microbiota to use host muco-glycoprotein as a nutrient source, leading to alteration of the intestinal barrier, partially composed of mucus in the colon [84]. This alteration is responsible for the invasion of enteric pathogens, like *Citrobacter rodentium*, leading to ulcerative colitis [84]. In addition, this low dietary fiber intake is responsible for dysbiosis, which leads to aggravation of intestinal barrier destabilization [84]. In an HS diet, chronic overconsumption leads to a loss of tight junction proteins in the proximal small intestine, a decrease in mucus thickness, and, finally, promotes systemic inflammation [85,86,87]. All these deleterious consequences are amplified by association with an HF diet [67,87,88], suggesting once more the negative effect on the health of the combination of HF and HS found in the WD.

The WD is associated with gut microbiota dysbiosis and impaired intestinal barrier integrity, which promote inflammation [58,59,60,61,62,63,64,65,66,67,68,69,70,71]. This process is partly induced by the translocation of bacterial components such as LPS, which strongly activate the immune system [33,61]. This exacerbated immune response contributes to both local intestinal inflammation and systemic low-grade inflammation [33,61]. In addition, pro-inflammatory cytokines and bacterial components may cross the BBB and promote neurological alterations [89,90,91].

### 4.3. Consequences of the Western Diet on the Brain

The WD has numerous negative effects on the brain, such as addictive-like eating behavior, chronic stress, anxiety, and depression through mechanisms involving the gut–brain axis, neuroinflammation, and HPA dysregulation [92]. However, although obesity is associated with neuropsychiatric disorders potentially driven by neuroinflammation, short-term consumption of WD has been shown to significantly impact cognitive functions in rodents even in the absence of an obese phenotype, suggesting that these effects may occur partially independently of obesity and could instead be linked to early diet-induced dysbiosis [93,94,95,96].

Using experimental models, several markers have been shown to be implicated in brain alterations under highly caloric diets. The brain-derived neurotrophic factor (BDNF), which plays a major role in stress regulation and mood disorders, is under-expressed in cognitive-related pathologies like Alzheimer’s disease [97] or depression [98], and also in cases of HF/HS diet consumption [99]. BDNF is linked to the cyclic AMP-responsive element-binding protein (CREB) pathway and synapsin 1 (SYN) to regulate synaptic plasticity, a mechanism related to brain health [100]. BDNF is mostly expressed in the hippocampus, where it is involved in learning and memory [100]. However, HF diet consumption is associated with a decrease in BDNF, SYN, and CREB levels and also the mRNA of growth-associated protein 43 (GAP-43), an important protein implicated in neurite growth [99,100]. In parallel, another negative impact of an HF diet is the increase in cerebral reactive oxygen species (ROS), which may also contribute to the decrease in BDNF, SYN, and CREB [101]. Concerning these neuronal markers, a reversal mechanism that could partially restore the neurotrophic factor BDNF levels concerns the positive effect of caloric restriction in obese patients [102].

Finally, increased levels of ROS are associated with alterations in spatial learning capacity, reduced spine density in the hippocampus and prefrontal cortex, and decreased long-term potentiation in the hippocampus [103,104,105]. Moreover, an HF diet has been associated with increased levels of malondialdehyde (MDA), a toxic molecule for neural progenitor cells, which may contribute to reduced neurogenesis, a process involved in maintaining a healthy neuronal network [106].

Furthermore, an HF diet alters the BBB, especially due to the presence of palmitic acid, which is responsible for decreased zonula-occludens-1 (ZO-1) and occludin, implicated in the tight junction of the BBB [107]. Disrupted BBB integrity is also found in neurodegenerative disorders, such as Alzheimer’s, Parkinson’s, and Huntington’s [57], and psychiatric disorders like schizophrenia or autism [53]. An increase in BBB permeability allows the penetration of pro-inflammatory cytokines and other metabolites, which can lead to dysregulation of brain functions and the development of neurological disorders, particularly through neuroinflammatory mechanisms [89,90,91].

Links between high-energy dietary patterns and brain dysfunctions have been demonstrated in experimental studies, and specific correlations have also been confirmed in humans. In rodents, consumption of an HF diet for 8 weeks induced morphological changes in microglia in the prefrontal cortex [103]. This modification leads to synapse pruning, which is finally responsible for synaptic loss [103]. Moreover, this study showed a decrease in corticosterone in obese rats, which appeared linked to a decrease in dendritic spine density in the prefrontal cortex [103]. The authors also showed a decrease in the cerebral volume, especially in the hippocampus, prefrontal cortex, and anterior cingulate [103], corresponding to brain regions involved in impulsivity, which can explain the increase in impulsive behaviors in obese patients [108]. As a parallel in humans, the alteration of the inhibition control in the prefrontal cortex leads to increased appetite and high-calorie dietary habits [109].

Telencephalic regions, such as the frontal cortex and hippocampus, appear particularly sensitive to WD consumption [110,111,112,113,114,115]. In an experimental model, Shi and colleagues showed that an HF diet induces early markers of neurodegenerative processes, especially in the hippocampus, including decreased tight junction protein expression, gliosis, and neuroinflammation [110]. The hippocampus is highly vulnerable to neuroinflammation and oxidative stress, which may ultimately contribute to synaptic dysfunction and cognitive impairment [111,112]. In mice subjected to an HF/fiber-deficient diet, the levels of SYN, synaptophysin (SYS), and post-synaptic density protein 95 (PSD95) were significantly reduced. These alterations, in these proteins playing a major role in synaptic plasticity and synaptogenesis, lead to morphological synaptic alterations and cognitive deficits [110]. These alterations have also been reported in patients with Alzheimer’s disease and other cognitive disorders [113,114,115].

Considering the acquisition of balanced nutritional habits, the young age appears particularly important. Adolescence is a key period for both nutrition and brain development, especially for the hippocampus, which is sensitive to HF and HS dietary patterns [116,117]. Consequently, overconsumption of sucrose by rats during this crucial developmental period leads to alterations in the adult reward circuit, resulting in altered motivated behaviors [118]. Additionally, several studies based on sugar overconsumption by rats during their adolescence presented less hedonic reactions in response to sweet tastes in adulthood [119]. This hedonic deficit is associated with lower c-Fos expression levels in the nucleus accumbens, a brain region known to play a central role in hedonic processing [119]. Anhedonia and motivational deficits have been reported as the hallmarks of several psychiatric disorders, including depression and schizophrenia, all emerging during adolescence [120]. Interestingly, chronic treatment with the antidepressant drug imipramine reversed all these deleterious consequences in hippocampal cells and acquired behaviors [121], suggesting a direct functional link between nutritional habits and neuronal circuits.

In summary, WD is a plague for industrialized countries, responsible for many comorbidities in cardiovascular, neurologic, or intestinal diseases [52,53,54,55,56,57]. The systemic inflammation, so-called “meta-inflammation”, induced by this type of feeding leads to a high exposition of the brain, gut, liver, adipose tissue, and pancreas to pro-inflammatory cytokines, such as TNFα, IL-6, and IL-1β [122,123]. This meta-inflammation can lead to intestinal dysbiosis, a leaky gut barrier, and the BBB, which can finally aggravate intestinal and neurological diseases. Cytokines are then considered as co-mediators between the brain and peripheral organs.

## 5. Consequences of the Mediterranean Diet on the Microbiota–Gut–Brain Axis

**Figure 3:**
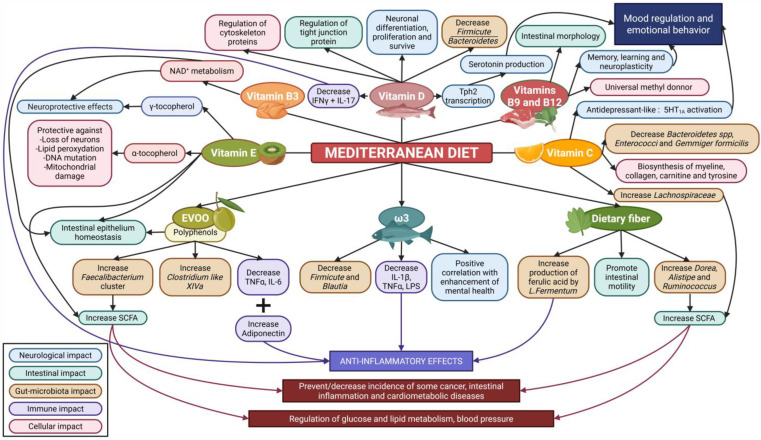
Impact of the main compounds of the Mediterranean diet on the microbiota–gut–brain axis. Non-exhaustive knowledge of relations established between compounds of balanced alimentation and direct/indirect consequences on the microbiota–gut–brain axis. The Mediterranean diet is characterized by a composition high in polyphenols, dietary fibers, ω-3, and vitamins. All those components, through potential synergic effects, may have a beneficial impact on the microbiota–gut–brain axis, with local antioxidant and anti-inflammatory effects, but also a broader impact through transcriptional and methylation regulation, for example. Created with BioRender.com (accessed on 27 March 2026) (SCFA: short-chain fatty acid, IL: interleukin, LPS: lipopolysaccharide, IFN: interferon, TNF: tumor necrosis factor, EVOO: extra virgin olive oil, 5HT1A: serotoninergic receptor 1A, Tph2: tryptophan hydroxylase 2).

The first time the MD was described was in the late 1950s, and the major characteristics were a diet poor in saturated fat and high in vegetable oil, such as olive oil, especially found in Greece, Spain, and Italy [124]. Since then, this definition has evolved over time, including the development of MD pyramids, graphical representations that illustrate the types and frequency of foods recommended for adherence to the MD pattern [125,126]. The consequences of MD consumption on the intestinal microbiota, the gut, and the brain are summarized in Figure 3.

According to scientific research in nutrition, MD is characterized by high consumption of fruits, vegetables, legumes, nuts, wholegrains, grains, aromatic herbs, and spices, and the use of extra virgin olive oil as the main source of fat. Moreover, highly aromatic herbs like parsley, oregano, mint, rosemary, thyme, coriander, and basil, as well as spices like cumin, cloves, saffron, cinnamon, and pepper, provide numerous antioxidant and anti-inflammatory properties for the organism [9,10,11,12]. In a review of studies on MD, Davis and colleagues described the composition of the MD based on 8 research papers: a mean of 9.3 ± 1.1 MJ/day, including 36.6 ± 4.9% energy of total fat with 18.8 ± 4.3% of monounsaturated fatty acids (MUFA), 4.8 ± 1% of polyunsaturated fatty acids (PUFA), and 9 ± 1% of saturated fat (SFA); 14.9 ± 2.3% of proteins ;and 42.8 ± 3.3% of energy from carbohydrates [125]. Finally, the beneficial effect of MD on human health can be explained by its nutritional value. MD is poor in SFA and animal proteins, rich in antioxidants, fibers, MUFA, probiotics, and monounsaturated fat, and provides a good balance between ω-6 and ω-3 [125,127].

In addition to macronutrients and complex bioactive compounds, a further distinguishing element of MD is vitamin intake, as described in Table 1. Vitamins correspond to a group of 13 organic compounds that are not endogenously synthesized but are essential for many physiological mechanisms, playing a key role as cofactors for metabolic and epigenetic regulation processes [128]. Consequently, vitamins can modulate the microbiota–gut–brain axis both directly and through interaction with the intestinal microbiota. Indeed, vitamins are mainly found in food and are essential at every stage of life [129]. A deficiency in even one vitamin can have disastrous consequences, whether at the embryonic or juvenile stages, with the risk of developmental disorders, but also at the adult stage, with the development of acute or chronic pathologies or the acceleration of the aging process [128,129,130,131,132,133].

A significant number of large-scale studies have been conducted on MD across different countries and physiological aspects, including impact on cardiovascular diseases, metabolic syndrome, colorectal cancer, and psychological disorders (Table 2) [149,150,151,152,153,154,155,156,157,158,159,160].

### 5.1. Consequences of the Mediterranean Diet on the Gut-Microbiota

Most of the beneficial effects of MD for health come from its impact on the intestinal microbiota. Indeed, Filippis and colleagues showed that MD plays a beneficial role in microbiota composition, thanks to the high level of consumption of plants [161]. People who follow an MD regime have a high proportion of SCFA and bacterial species that can degrade the dietary fibers, thanks to the intestinal microbiota [161]. Other authors confirm the positive impact of MD on the intestinal microbiota profile, given that people with high adherence to the MD regime have a lower presence of *E. coli* and an increase in total bacterial abundance, along with a change in specific taxa (e.g., increased *Bifidobacteria*/*E. coli* ratio), and an enhanced SCFA production [162,163]. Consequently, MD has positive effects on the composition of intestinal microbiota, mainly in richness or evenness (named α-diversity) and metabolic activity [162,163]. The high production of SCFA contributes to preventing or reducing the incidence of some cancers (especially colorectal cancer), intestinal inflammation, and cardiovascular-metabolic pathologies [164,165]. Concerning bacterial species, a positive correlation has been shown between polyphenols in MD and the presence of specific *Clostridium*-like XIVa and *Faecalibacterium* clusters, which synthesize butyrate and are involved in the anti-inflammatory effects of MD [162,166].

In a diabetic rat model, the administration of flaxseed oil, rich in α-linolenic acid, improves glucose metabolism, decreases IL-1β, TNFα, and MDA levels, and increases SCFA production compared to the control group [167]. The administration of flaxseed oil increases the relative abundance of *Bacteroidetes* and *Alistipes* and decreases the relative abundance of *Firmicutes* and *Blautia* [167]. Finally, supplementation with flaxseed oil leads to modifications in the intestinal microbiota in favor of SCFA production, which decreases inflammation, as indicated by decreased levels of pro-inflammatory molecules IL-1β, TNFα, and LPS, caused by diabetes [167]. Dietary fibers play a prebiotic role in microbial growth in the human intestines. In fermentable fibers, oligosaccharides, β-glycan, and cellulose are very good substrates for bacterial SCFAs production [168]. According to Nicholson and colleagues, these SCFAs physiologically connect the gut microbiome to other organs, such as the brain, with immunity regulation, glucose and lipid metabolism, for positive consequences [169]. Dietary fibers can also stimulate the production of ferulic acid (FA) by *L. Fermentum*, which has antioxidant and anti-inflammatory functions, and an “anti-diabetic” effect was shown in male rats: FA normalized serum insulin levels [170].

Focusing on specific compounds of MD, extra virgin olive oil (EVOO) contains polyphenols, a secondary metabolite of plants derived from phenylalanine and tyrosine, and oleic acid [171]. These two compounds exert antioxidant and anti-inflammatory roles, with a direct impact during intestinal absorption or an indirect impact after transformation in the gastrointestinal tracts [172]. They induce the production of specific metabolites, such as hydroxytyrosol, which can modulate the biological response of the host, especially concerning the activation of antioxidant enzymes through the Nrf2 transcription factor, and also reduce pro-inflammatory cytokines while increasing anti-inflammatory ones [173].

A 3-month monitoring study in 18 obese or overweight patients vs. 18 controls showed that MD with 40 g/day of EVOO increases the composition of the intestinal microbiota in lactic acid bacteria, which contribute to increased levels of the anti-inflammatory cytokine IL-10 [174].

A molecular variant of vitamin E, γ-tocopherol, was reported to impact the intestinal microbiota by inducing a depletion of *Roseburia* [175]. *Roseburia hominis* is a butyrate producer and is decreased in the feces of ulcerative colitis patients [176]. Butyrate, as stated above, is an SCFA and can consequently influence the brain [46,49,50].

In a study in which 14 healthy participants received a high-dose ascorbic acid supplementation for two weeks (1000 mg/day), vitamin C was found to affect both the intestine and gut–microbiota, with a significant increase in relative abundances of *Lachnospiraceae* and a decrease in those of *Bacteroidetes, Enterococci*, and *Gemmiger formicilis* [177]. However, in patients with liver cirrhosis, an increase in the genus *Enterococcus* was associated with inflammation and a reduction in cognitive functions [178]. *Enterococcus* and *Gemmiger formicilis* are correlated with Crohn’s disease relapse [179]. Moreover, the *Lachnospiraceae* family belongs to the phylum *Firmicutes*, which is predominant in healthy people [180], and its relative abundance is decreased in patients with Crohn’s disease [181]. *Lachnospiraceae* is one of the main producers of SCFAs, which have antioxidant and anti-inflammatory properties [45]. Thus, vitamin C appears to alter gut microbiota in a manner that may be beneficial to the host. However, the study by Otten, mentioned above, included only 14 participants and used high doses of ascorbic acid; therefore, larger-scale and longer-term studies are needed to confirm a beneficial conclusion.

In humans, a cross-sectional study conducted in individuals with an endoscopically normal colon found that lower consumption of vitamins B9 and B12 was associated with significant modifications of the gut microbiota, including decreases in richness and evenness, alterations in β-diversity, and modifications in the relative abundance, such as a decrease in *Odoribacter* [182]. *Odoribacter splanchnus* is known to produce SCFAs, which are involved in the modulation of inflammation and also in epigenetic programming [183,184]. Consequently, lower consumption of vitamins B9 and B12 may lead to alterations in the intestinal microbiota, potentially contributing to a decrease in SCFA production and to the deleterious consequences described above. Moreover, vitamin D may maintain the diversity of the intestinal microbiota [185,186].

### 5.2. Consequences of the Mediterranean Diet on Gut

A study with 142 IBD patients undergoing a short 180-day dietary intervention based on an MD showed a significant reduction in malnutrition-related parameters, associated with the reversion of inflammatory markers [187]. Concerning EVOO, the authors showed an impact on the intestines, especially through phenolic compounds, which are responsible for beneficial modulation of intestinal epithelium homeostasis [187]. Several studies have shown that EVOO can help modulate intestinal inflammation and immune response and reduce oxidative stress, thus preventing the onset of inflammatory or degenerative diseases [173]. In addition, a study in 18 overweight patients showed that MD with EVOO supplementation (40 g/day) led to improved anti-inflammatory effects of this diet, with a reduction in various markers: myeloperoxidase (inflammation and endothelial dysfunction); 8-hydroxy-2-deoxy-guanosine (oxidative DNA damage); TNFα and IL-6 (pro-inflammation); but also an increase in adiponectin (regulation of the expression of the anti-inflammatory IL-10) [174].

In mice receiving a dose of α and γ tocopherol equivalent to a daily intake of 300 mg for a 60 kg adult, Liu and colleagues showed a decrease in colitis severity, with attenuation of diarrhea and fecal bleeding, as well as inhibition of colonic pro-inflammatory cytokine expression [175]. Moreover, vitamin D intake has consequences for intestinal barrier integrity, especially through the regulation of tight junction expression [188,189]. In addition, vitamin D is involved in immunomodulation by decreasing both IFNγ and IL-17 [190,191].

Bressenot and colleagues showed that a deficiency in vitamins B9 and B12 during gestation and lactation in an animal model induced several morphological changes in the distal small intestine, including hypertrophy, decreased villi thickness, reduced crypt size, and changes in submucosal thickness [130]. Deficiencies in these two vitamins have been linked to IBD in humans [192,193]. In contrast, methyl donor supplementation in an experimental model of Crohn’s disease was shown to decrease the intestinal inflammatory marker calprotectin, increase the anti-microbial peptide levels, and prevent intestinal pathogenic colonization [194].

### 5.3. Consequences of the Mediterranean Diet on the Brain

Numerous epidemiological studies have tested the MD in psychological disorders [195,196,197]. The PREDIDEP study was based on the MD supplemented with EVOO to prevent the risk of relapse of unipolar depression after 2 years of monitoring [195]. This study used much information, especially quality of life, physical activity, dietary habits, blood, urine, and fecal monitoring, and provided knowledge concerning the impact of MD for preventing the recurrence of depression [195]. In the SMILES trial, Jacka and colleagues evaluated the effectiveness of MD as a treatment for 67 subjects with major depressive disorder, including 72% women [196]. In comparison with a control group, the group subjected to 12 weeks of MD showed significant improvement according to the “Montgomery–Åsberg Depression Rating Scale”. Finally, remission was observed in 32% of patients in the MD group [196]. Another trial was HELFIMED, in which the authors tested the effect of the MD supplemented with fish oil in 152 subjects (30.9% men and 69.1% women) with depressive symptoms [197]. The group in which the intervention was performed received MD cooking workshops for 3 months and fish oil supplementation for 6 months. After 3 months, the MD groups had the best adherence to the MD regimen, with more consumption of vegetables, fruits, nuts, and legumes, and fewer unhealthy snacks, red meat, and chicken. Consequently, the MD group presented a decrease in depressive symptoms, with an improvement in mental health score [197]. The decrease in depression was significantly correlated with an increase in adherence to MD and consumption of nuts and vegetables. Moreover, this study showed a positive correlation between the increase in ω-3, the decrease in ω-6, and the enhancement of mental health [197].

The MD also contains fish, seafood, and nuts, which are rich in ω-3 fatty acids that contribute to balancing ω-6 fatty acids and may consequently lead to reduced inflammation [198]. Correlations between PUFAs and brain health have also been reported in humans, with lower ω-3 fatty acid intake observed in cases of cognitive decline, including Alzheimer’s disease and dementia [199]. In addition, ω-3 PUFA consumption was shown to improve depressive symptoms and quality of life in elderly women with depression [200]. It has been recently shown that ω-3 fatty acids can be metabolized in brain cells through the lipoxygenase and cytochrome P450 enzymes, leading to reduced apoptosis, and promoting neurogenesis in hippocampal cell lines, while also correlating with less severe depressive symptoms in patients [201].

Vitamin E, in the form of α-tocopherol, had a protective effect against lipid peroxidation [202], DNA mutations [203,204], mitochondrial damage [205], neuronal loss [206], and amyloid-β deposition [207]. In humans, Morris and colleagues showed that α and γ tocopherol intake was significantly associated with a lower rate of cognitive decline and also a lower incidence of Alzheimer’s disease [208]. More specifically, another study investigating the interaction between α- and γ-tocopherol in Alzheimer’s disease suggested that a high dose of α-tocopherol alone does not provide neuroprotection [208]. Amyloid-β levels were lower when both α- and γ-tocopherol concentrations were high, suggesting that their interaction may be necessary for the neuroprotective effect observed in Alzheimer’s disease [208]. A systematic review and meta-analysis of vitamin E supplementation reported inconclusive results regarding the treatment of both depression and anxiety [209]. However, the beneficial effects of vitamin E intervention appear to depend on the study context. For example, Malaguarnera and colleagues showed a significant decrease in depression and anxiety in patients with chronic hepatitis C treated with pegylated interferon and ribavirin and supplemented with 30 mg/day of vitamin E [210]. In contrast, another study showed no significant improvement in depression among patients with amnestic mild cognitive impairment receiving vitamin E supplementation [211]. Nevertheless, considering that psychiatric syndromes can have multiple etiologies and human cohorts’ heterogeneity, such variability may contribute to inconsistent findings across studies.

Vitamin C plays a fundamental role in the brain, contributing to antioxidant defenses and to the biosynthesis of collagen, carnitine, tyrosine, peptide hormones, and myelin [212]. Moreover, ascorbic acid is essential for neurotransmission and neuronal maturation and functions [213]. Many studies have described the roles of vitamin C in neurodegenerative diseases such as Alzheimer’s and Parkinson’s diseases, as well as in psychiatric disorders including depression, anxiety, and schizophrenia [214,215,216,217,218]. Indeed, vitamin C has been reported to exert antidepressant-like effects through activation of the serotonin 1A receptor (5-HT1A) [219].

Vitamin D, which is locally synthesized by neurons and microglia, is involved in the regulation of differentiation, proliferation, and cell survival [220]. The synaptic plasticity and molecular transport of cell organelles also depend on vitamin D, which regulates the expression of many proteins involved in cytoskeletal maintenance (e.g., tubulin and MAP-2) [221]. The vitamin D receptor (VDR), which has been widely identified in the limbic system (e.g., in the hippocampus and the prefrontal cortex), is involved in mood regulation and emotional behavior [222]. Furthermore, vitamin D activates the transcription of tryptophan hydroxylase 2 (Tph2), an enzyme implicated in the conversion of tryptophan to serotonin, and thus contributes to the regulation of serotonin synthesis [223]. Accordingly, given these major roles, vitamin D appears to be involved in several neurological disorders, including schizophrenia, autism spectrum disorder, Parkinson’s, and Alzheimer’s diseases [224].

In 1970, a first publication highlighted a link between depression and plasmatic folate concentration, which was much lower in individuals with depression than in those without psychiatric disorders [225]. Since then, it has been shown that hypovitaminosis in B9/B12 is directly associated with elevated homocysteine levels (i.e., the cytotoxic molecule commonly used as a marker) and decreased levels of S-adenosyl-methionine (SAM) (i.e., the universal cellular methyl donor), leading to alterations in methylation reactions and in the metabolism of neurotransmitters such as dopamine, noradrenaline, and serotonin [226]. In addition, several studies have shown that methyl donor deficiency and the resulting hyperhomocysteinemia are risk factors for the development of neurodegenerative diseases [227]. Indeed, aggregation of several proteins through N-Homocysteinylation has been shown to impair the balance between neuronal proliferation and differentiation, thereby affecting neuroplasticity [228]. It has also been reported that dysregulation of the folate and cobalamin cycles affects the glucocorticoid response in the hypothalamus through post-translational modification of the glucocorticoid receptor [132]. Moreover, methyl donor deficiency during early development results in decreased expression of glutamatergic NMDA receptors and of the associated postsynaptic stabilizing protein PSD95 in the CA1 region of the hippocampus, resulting in impaired hippocampal-dependent memory [229]. Finally, deficiencies in vitamins B9 and B12 have been linked to Alzheimer’s disease [230].

### 5.4. Synergistic Effects of Micronutrients Included in the Mediterranean Diet

Although individual components of the MD exhibit specific beneficial properties as described above, it is essential to consider the synergistic effects associated with their simultaneous consumption. This synergy contributes to antioxidant and anti-inflammatory processes, as described above and illustrated by the interactions between vitamins E and C, since vitamin E plays a major antioxidant role [231] and interacts with other antioxidants such as vitamin C and glutathione to recover its non-oxidized state [232]. As a more indirect synergy, polyphenols (i.e., flavonoids) may influence intestinal microbiota composition, with an increase in relative abundance of *Bifidobacterium* and *Lactobacillus* [233,234], which are able to produce and influence some B vitamin groups such as B6, B9, or B12 [235]. Then, polyphenols may indirectly influence the availability of B vitamins. Beyond these effects, such synergy may also extend to broader regulatory mechanisms, particularly methylation processes and transcriptional regulation.

Vitamins B9 and B12 are essential for epigenetic regulations since they are involved in the folate and methionine cycles, which are required for the transfer of methyl groups to several molecules, including nucleic acids, but also for regulating proteins (e.g., histones or transcription factors) [128]. The methionine cycle serves to re-methylate a cytotoxic amino acid, homocysteine, into methionine using the folate cycle and proposes a methyl group with the SAM for trans-methylation reactions [128]. Consequently, folate and cobalamin are called “methyl group donors”, and these two essential vitamins are implicated in many aspects of metabolism and physiology, as shown in the previous section [236].

In parallel, polyphenols found in EVOO have been associated with the prevention of DNA damage, inhibition of the proliferation of breast, prostate, and colon cancer cells, and modulation of the activity of several histone deacetylases (HDAC) [237,238]. Moreover, the complex formed by active vitamin D and VDR acts as a transcription factor involved in the regulation of the expression of more than 900 genes implicated in numerous physiological functions [239], including immune and inflammatory responses [190,191], cellular proliferation and differentiation [220], behavior [222,223], and intestinal barrier integrity [188,189].

Finally, these non-exhaustive mechanisms should be considered as part of a complementary network rather than isolated processes, since B vitamins support methylation capacity, polyphenols modulate epigenetic enzyme activity, and vitamin D regulates gene transcription. These multiple regulatory levels, which also extend to antioxidant and anti-inflammatory effects, highlight the importance of synergistic interactions in the beneficial outcomes associated with the MD. However, human trials rarely isolate true interaction effects; therefore, although the impact of this synergy on methylation is biologically plausible, it remains poorly quantified.

## 6. Conclusions

From early development to aging, nutrition plays a major role in shaping metabolism and physiology. As described in this review, WD induces deleterious effects, notably through the installation of gut microbiota dysbiosis, altered intestinal permeability, and local and systemic inflammation. Recent studies have linked these peripheral modifications to brain structural and functional consequences that may contribute to neurological disorders in so-called gut–brain axis communication. As we summarized here, deficiencies in one or more vitamins, an imbalance between ω-3 and ω-6, or low fiber intake related to unsuitable diets are closely associated with these alterations and may contribute to the development of metabolic, intestinal, neurological, and neuropsychiatric disorders.

In terms of reversibility, experimental model evidence suggests that WD-induced alterations may persist despite dietary normalization, with reports of sustained cecal dysbiosis, dopaminergic sensitization associated with increased vulnerability to compulsive alcohol consumption, and perpetuation of epithelial and hepatic dysfunction [240].

In this context, supplementation with microbiota-accessible carbohydrate (MAC) (green shield in Figure 2) may represent an intermediate strategy to counteract WD-induced alterations. These non-digestible carbohydrates, derived from plant fibers, are fermented by the gut microbiota and promote the production of bioactive metabolites such as SCFA [241,242,243]. MAC supplementation in the WD context prevents dysbiosis, leaky gut, systemic inflammation, and microglia activation [110]. Specific MACs, such as inulin and resistant starch, have been shown to modulate gut microbiota composition, promoting a shift from proteolytic to saccharolytic fermentation, which is associated with improvements in glucose metabolism and intestinal homeostasis [244,245]. However, the effects of MAC are not uniform, as highly fermentable compounds, such as inulin, may induce gastrointestinal side effects in sensitive individuals, such as irritable bowel syndrome patients [246]. This highlights the need to consider inter-individual variability when implementing such dietary strategies.

Adopting balanced dietary patterns such as the MD appears to be one of the most effective strategies to mitigate WD-associated alterations. Rather than considering diet as acting on a single organ, it should be viewed as a complex, systemic modulator interacting with multiple interconnected networks, extending beyond its initial impact on the gut microbiota. While most studies have focused on the direct effects of diet on the host, significant gaps remain in understanding the mechanisms underlying early-life programming, particularly regarding maternal influences and potential microbial transfer to offspring. Although no specific nutritional guidelines are currently established for some pathologies such as IBD, the MD—rich in fiber, polyphenols, and omega-3 fatty acids—emerges as a low-risk and accessible strategy to support the microbiota–gut–brain axis across the lifespan.

## Figures and Tables

**Table 1:** Daily vitamin intakes recommended and provided by the Mediterranean diet.

Vitamins	Recommended	Mediterranean Diet	Prevalent Dietary Source
D, ^1^ µg/d	20.00 [134]	0.30–3.80 [135]	Fatty fish like salmon or tuna, dairy products [136].
E, mg/d	15 [137]	17.00 [138]	Olive oil or other vegetable oils, nuts, fruits like kiwi, and some fish [139].
C, mg/d	100–200 [140]	137.20–269.81 [125]	Citrus fruits, tomatoes, red peppers, and Brussels sprouts [141].
A, ^2^ µg/d	300–1300 [142]	1273.3 [143]	Liver, fish, eggs, and dairy products [144].
B12, µg/d	0.9–2.8 [142]	1.50–2.00 [145]	Meat, fish, milk, and eggs [146].
B9, µg/d	150–600 [142]	400 [145]	Beef liver, spinach, rice, asparagus, lettuce, and avocado [147].

^1^ Vitamin D as cholecalciferol, 1 µg cholecalciferol = 40 IU vitamin D, assuming minimal sunlight exposure [142]. ^2^ Retinol activity equivalents = 1 µg retinol, 12 µg β-carotene, 24 µg α-carotene, or 24 µg β-cryptoxanthin [142]. Table adapted from [148].

**Table 2:** Summary of studies about the Mediterranean Diet and its benefits ont health.

Name	Date	Countries	Study Design	Primary Outcomes	Main Limitations	References
Seven countries study	First phase: 1958–1983 Second phase: 1984–1999	Italy, Finland, Greece, United States, Yugoslavia (Croatia and Serbia), Japan, and Netherlands	Prospective cohort—16 cohorts of 12,763 middle-aged men with lifestyle and dietary habits monitoring for up to 25 years.	MD adopted in Greece and Italy. MD not adopted in Northern Europe and the United States. Lower cardiovascular mortality in the Mediterranean population. Suggest prevention of cardiovascular diseases by decreasing fat consumption.	Observational only, men-only, middle-aged-only, rural population focus. Missing specific distinction: bread and cereals rich *vs* poor in fiber; fresh *vs* dried fruit; olive oils *vs* other oils; red meat *vs* other types of meat; fat fish *vs* lean fish; whole milk *vs* skim milk; fat *vs* lean dairy products.	[149,150]
EPIC Study (European Prospective Investigation into Cancer and nutrition)	1993–1999	Denmark, France, Germany, Greece, Italy, Holland, Spain, Norway, United Kingdom, and Sweden	Prospective cohort—519,978 subjects regularly contacted with 3 to 5 years intervals, to evaluate their lifestyle.	The aim of this study was to evaluate the links between nutrition, environment, lifestyle, and the incidence of cancers and other chronic diseases. MD is considered the most effective alimentation model for cancer prevention. Moreover, regular yogurt consumption is associated with a decreased incidence of colorectal cancer, likely due to the probiotic benefits it provides.	Only observational, Confounding lifestyle bias.	[151,152]
PREDIMED (Prevencion con Dieta MEDiterrannea) multicenter study	2003–2010	Spain	Randomized controlled trial—7447 subjects randomized following an MD with supplementation in olive oil, or nut *vs* a control diet.	This study describes and assesses the long-term effect of MD in cardiovascular disease and other clinical conditions, such as Alzheimer’s.	Retraction of the study prior to republication, due to randomization issues. Only one country generalized to the global population. Specific population with a high risk of cardiovascular disease. Synergy between all components of MD: complicated to determine which micronutrient is responsible for the observed modification.	[153,154,155,156]
PREDIMED-PLUS study	2013–2016	Spain	Randomized controlled trial—6874 patients recruited in 23 hospitals	The aim of this study was to evaluate the impact of lifestyle (physical activity) and MD in the prevention of cardiovascular disease. Physical activity and MD were significantly associated with improvements in components in metabolic syndrome, triglycerides, inflammatory markers and blood cholesterol.	Only one country generalized to the global population. Specific population with a high risk of cardiovascular disease. Long-term consequences are still under evaluation.	[157,158,159,160]

## Data Availability

No new data were created or analyzed in this study. Data sharing is not applicable to this article.

## Abbreviations

The following abbreviations are used in this manuscript:

5HT1A	Serotonin 1A receptor
ACTH	Adrenocorticotropic hormone
BBB	Blood–brain barrier
BDNF	Brain-derived neurotrophic factor
CNS	Central nervous system
CREB	Cyclic AMP-responsive element-binding protein
CRF	Corticotropin-releasing factor
EVOO	Extra virgin olive oil
F/B	Firmicutes/bacteroides
FA	Ferulic acid
GAP-43	Growth-associated protein 43
GPR43	G protein-coupled receptor 43
H_2_S	Hydrogen sulfide
HDAC	Histone deacetylase
HF	High-fat
HPA	Hypothalamic–pituitary–adrenal axis
HS	High-sucrose
IBD	Inflammatory bowel disease
LPS	Lipopolysaccharide
MD	Mediterranean diet
MDA	malondialdehyde
MUFA	Monounsaturated fatty acids
NTD	Neural tube defects
PSD95	Post-synaptic density protein 95
PUFA	Polyunsaturated fatty acids
PVN	Paraventricular nucleus
ROS	Reactive oxygen species
RS	Resistant starch
SAM	S-adenosyl-methionine
SCFA	Short-chain fatty acid
SFA	Saturated fat
SYN	Synapsin I
SYS	Synaptophysin
TLR4	Toll-like receptor 4
TPH2	Tryptophan hydroxylase 2
VDR	Vitamin D receptor
WD	Western diet
ZO-1	Zonula-occludens-1
